# The semantics of person and *de se* effects in free indirect discourse

**DOI:** 10.1186/s40064-016-3102-8

**Published:** 2016-08-30

**Authors:** Denis Delfitto, Gaetano Fiorin, Anne Reboul

**Affiliations:** 1 University of Verona, Via dell’Artigliere 4, 37129 Verona, Italy; 2 University College Utrecht, Utrecht, The Netherlands; 3 Institute for Cognitive Sciences-Marc Jeannerod, CNRS UMR5304, Lyon, France

**Keywords:** Free indirect discourse, Personal pronouns, Context of utterance, Context of thought, Phenomenal identification, De se

## Abstract

In this contribution we will address the main puzzling empirical issues that have been formulated around Free Indirect Discourse (FID): the constraints on the use of first person pronouns and of proper names (as well as of definite descriptions), the reasons why different grammatical features (person, gender, number) give rise to presuppositions that must be resolved at different levels of interpretation in FID, the factors that account for the observation that person and tense behave similarly in FID. At the same time, we will also discuss the main controversies to which the ongoing debate on FID has given rise in the literature, showing that Schlenker (Mind Lang 19(3):279–304, 2004)’s distinction between a Context of Thought (CT) and a Context of Utterance (CU) still provides a fundamentally valid insight into the nature of FID, in spite of many qualifications that are necessary and some well-motivated criticism. However, our main task here is more ambitious than simply taking a stand on the many unsettled controversies surrounding FID. In fact, we claim that Schlenker’s split between CU and CT can be derived in a principled way from the inner nature of FID as a linguistic process of ‘phenomenal identification’, whereby a Higher Experiencer attempts at reproducing (at a distinct time) the phenomenal experience proper to a Lower Experiencer. This distinction between qualitatively identified but numerically distinct experiences provides the conceptual basis for the derivation of virtually all remarkable properties of FID (including its somehow intermediate status between Direct and Indirect Discourse), while connecting, at the same time, with some intriguing semantic properties of first-person pronouns, such as the different varieties of *de se* readings.

## Background

FID is a narrative style that is used to report the words or thoughts of a character.^1^ Its correct semantic characterization as well as its precise status with respect to direct and indirect discourse is still largely debated. Banfield (1982) notices that FID displays features of both types of discourse.^2^ Consider the following example:

1. Tomorrow was Monday, Monday, the beginning of another school week! (Lawrence, *Women in Love*, p. 185, London Heinemann 1971; example used by Banfield (1982, p. 98)

As in direct discourse, the narrator is reporting the perspective of the protagonist. This is particularly clear in the use of the temporal indexical *tomorrow*, which, in the reporting FID, clearly is understood relatively to the temporal perspective of the character whose thoughts are reported. In this respect, FID behaves as a direct report.

Furthermore, FID is ‘free’ in that it needs not be introduced by a superordinate clause, such as *x said/thought that*, although a framing expression, such as *x said/thought*, can be added as a parenthetical:

(2) Tomorrow was Monday, she thought/said, Monday, the beginning of another school week!

It seems, therefore, that most features of the discourse or thought reported are preserved in FID, similarly to what happens with direct discourse.

However, contrary to what we find in direct discourse, tense and pronouns reflect the narrator’s perspective, as would be the case in indirect discourse.^3^ For example, the past tense *was* in (1) is evaluated relatively to the narrator’s perspective, as it would be in indirect discourse; unless we adopt a dual-voice account, where both the perspectives of the author and those of the character are represented, this would be paradoxical.

The following set of examples further elucidates the pattern we have described:

(3) a. *Direct discourse:* Mary thought, ‘**Tomorrow***is my* birthday.’
  b. *Indirect discourse:* Mary thought that **the next day***was her* birthday.
  c. *FID:***Tomorrow***was her* birthday, Mary thought.

Notice in particular how, in FID, *tomorrow* is maintained as in a direct report, whereas the present tense and first person pronoun are modified as if they were occurring in an indirect report. Thus there are quite a few puzzles about FID.

In this contribution we will address the main puzzling empirical issues that have been identified around Free Indirect Discourse (FID): the constraints on the use of first-person pronouns and of proper names (as well as of definite descriptions), the reasons why different grammatical features (person, gender, number) give rise to presuppositions that must be resolved at different levels of interpretation in FID, the factors that account for the observation that person and tense behave similarly in FID. At the same time, we will also discuss the main controversies to which the ongoing debate on FID has given rise in the literature, showing that Schlenker (2004)’s distinction between a Context of Thought (CT) and a Context of Utterance (CU) still provides a fundamentally valid insight into the nature of FID, in spite of many qualifications that are necessary and some well-motivated criticisms. However, our main task here is more ambitious than simply taking a stand on the many unsettled controversies surrounding FID. In fact, we claim that Schlenker’s split between CU and CT can be derived in a principled way from the inner nature of FID as a linguistic process of ‘phenomenal identification’, whereby a Higher Experiencer attempts at reproducing (at a distinct time) the phenomenal experience proper to a Lower Experiencer. This distinction between qualitatively identified but numerically distinct experiences provides the conceptual basis for the derivation of virtually all remarkable properties of FID (including its somehow intermediate status between Direct and Indirect Discourse), while connecting, at the same time, with some intriguing semantic properties of first-person pronouns, such as the different varieties of *de se* readings. The paper is organized as follows. In the first and second sections, we critically discuss some of the previous approaches to FID, concentrating on the contrasting proposals in Schlenker (2004) and Maier (2015). In the third section, we will present the main features of our analysis of FID as a linguistic process of phenomenal identification, by emphasizing both its cognitive and linguistic relevance. At the end, we will provide a short summary of the main results achieved.

## Two analyses of FID

In this section, we present two analyses of FID that we believe are representative of two main different classes of approaches to FID. We first present Schlenker (2004), as the most significant representative of those approaches [primarily developing ideas introduced in Banfield (1982)] that regard FID as a result of some operation of context shift. We then consider the most relevant criticisms formulated against the context shift approach [in particular, the criticisms put forward by Maier (2015) and Reboul (2013)]. We finally present the analysis of Maier (2015), as representative of those approaches that regard FID as a case of mixed quotation.

### FID as context shift: Schlenker (2004)

Schlenker (2004) uses FID as the basis for proposing a distinction between two types of contexts of evaluation, *context of thought* and *context of utterance*, and two corresponding types of indexicality: (a) ‘Referential’ indexicals and demonstratives, which receive their denotation from the context of thought; (b) pronouns and tenses, which depend on the context of utterance. Schlenker further suggests that there may be a principled reason for the proposed distinction. The meaning of ‘referential’ indexicals and demonstratives is lexically specified and depends on the intentions of the speaker/thinker; such expressions are hence evaluated relatively to the context of *thought*. On the other hand, pronouns and tenses are assumed to be variables whose domain of reference is restricted by grammatical features, such as gender, person, and tense; such features “serve as a system of classification whose referential domain is the utterance itself” (Schlenker 2004, p. 280); hence, their dependency on the context of *utterance*.

Schlenker (2004) shows that the distinction he proposes can also account for another reporting style, the historical present. Whereas in FID the actual context is identified with the context of utterance and the context of thought corresponds to that of the reported speech or thought, in the historical present the opposite applies: the context of thought is identified with the actual context whereas the context of utterance is that of the reported speech or thought. The historical present is, Schlenker contends, one of the combinatorial possibilities expected once the distinction between context of thought and context of utterance is made.

### Arguments against Schlenker (2004)

#### Maier (2015)

Maier (2015) discusses two main arguments against Schlenker’s proposal. In particular, with reference to the pronominal domain, Maier provides evidence that in FID (i) not all pronouns are evaluated relative to the context of utterance and (ii) not only pronouns are evaluated relative to the context of utterance.

Evidence for the first conclusion comes from the interpretation of the gender features of third person pronouns in FID [a problem already observed by Schlenker (2004), following Doron (1990)]. Consider the following example:

(4) [Mary wrongly believed that Robin was male. In fact, Robin was a woman.]
  a. Where was he this morning, for instance? (Mary wondered.)
  (Example from Schlenker 2004, p. 291)
  b. #Where was she this morning, for instance? (Mary wondered.)

The felicitous use of *he* and the infelicitous use of *she* in the examples above show that the gender features of the pronouns depend on the protagonist’s perspective, rather than on the narrator’s perspective. In Schlenker’s system, this would mean that they are evaluated relative to the context of thought, and not relative to the context of utterance. This fact contradicts Schlenker’s claim that pronouns are always interpreted relatively to the context of utterance; in fact, at least their gender features must be evaluated relative to the perspective of the author of the reported speech or thought. Schlenker (2004, p. 291) tentatively suggests, as a possible solution to this issue, that *he* in the example above is a pronoun of laziness, standing for an elided definite description. However, Maier rightly suggests that this is more of a stipulation than of a solution.

Evidence for the second conclusion comes from the observation that a ‘transparency effect’ (that is, evaluation in the context of utterance) is often achieved by replacing a second- or third-person pronoun with a proper name. Consider the following text fragment (from Maier 2015):

(5) Bill and Eric were fighting, when Sookie stepped between them. **Did Bill really think he could challenge his boss like that?** she demanded, before turning to Eric. **And what the hell was HE thinking?**

Here, we adopt the convention of signaling FID by putting the relevant portions of text in bold. Notice that the first occurrence of “Bill” in FID (as well as the third-person pronoun that immediately follows) stands for a mapping from a second-person to a name, since what Sookie actually utters in the context of thought is a sentence such as “Do *you* really think you can challenge your boss like that?”. Notice also that in the second fragment of FID the third-person *he* also stands for a mapping from a second-person to a name, since mapping FID into direct discourse would produce here an output along the lines of “And what the hell are *you* thinking?”

These observations clearly warrant the conclusion that transparency is not limited to pronouns, it also extends to names. At the same time, they raise the question why a second-person pronoun is sometimes shifted into a third-person pronoun and sometimes shifted into a name.

#### Reboul (2013)

Reboul also presents some potentially decisive arguments against Schlenker’s view that pronouns are necessarily interpreted relatively to the context of utterance in FID. Consider the following fragment from Flaubert’s *L’Éducation sentimentale*:

(6) Before he left for the holidays, he [Frédéric] had the idea of a picnic, to close the Saturday meetings. He was cheerful. **Mme Arnoux was at her mother’s in Chartres. But*he*would soon meet her again and would succeed at becoming her lover.**

In fact, we know that Frédéric becomes Mme Arnoux’s lover only in the context of thought, which is different from the real context, whereas in the context of utterance, the real context, Frédéric never becomes Mme Arnoux’s lover. So, it must be the case that *he* in (6) refers to the Frédéric of the context of thought, rather than to the Frédéric of the context of utterance. This entails that there are contexts when the referent of a pronoun is picked up in the context of thought and not in the context of utterance—*pace* Schlenker.

Reboul also offers a version of the same argument involving the first- rather than the third-person. Consider the fragment in (6), from Modiano’s novel *Accident nocturne*:

(6) I drew out of my pocket the “report” I had signed. So she was living in the square de l’Alboni. I knew that place because I had often got down at the nearest underground station. No problem that the number was missing. **With the name: Jacqueline Beausergent, I would manage**

In this novel, the protagonist (who is also the narrator, that is, Modiano) is looking for a young woman who has run him down in her car. The address on the “report” is incomplete, but the protagonist thinks he will “manage” to find her nevertheless. However, in the novel, he does not find the young woman and the narrator (the present time Modiano who is writing the story) knows that he will not. In other words, we have here a situation in which Modiano (the referent of the first-person pronoun “I” in the FID fragment) finds Jacqueline Beausergent only relatively to the context of thought and, crucially, not relatively to the context of utterance. It must thus be the case that “I” in (6) refers to the Modiano of the context of thought, and not to the Modiano of the context of utterance. The use of the first-person pronoun in (6) exactly parallels the use of the third-person pronoun in (5), since these are occurrences in which the pronoun is interpreted relatively to the context of thought and not relatively to the context of utterance, as predicted by Schlenker.

### FID as mixed quotation: Maier (2015)

Maier’s proposal is that FID represents a form of ‘mixed quotation’, with a strong prevalence of linguistic main clause indicators (direct questions, discourse particles and expressive elements reflecting the protagonist’s perspective, etc.) and metalinguistic main clause indicators (typographical elements, shift to dialect, etc.). As an example, Maier proposes the logical form in (7b) for a FID such as (7a).

(7) a. Ashley was lying in bed freaking out. Tomorrow was her 6 year anniversary with Spencer and it had been the best 6 years of her life.
  b. Ashley was lying in bed freaking out. **‘**Tomorrow **[**was**] [**her**]** 6 year anniversary with Spencer and it **[**had**]** been the best 6 years of **[**her**]** life**’**.

The analysis is based on two chief ingredients: First, a mechanism of quoting, represented in (7b) as ‘…’, which indicates that the expressions occurring inside it are mentioned—they refer to themselves *qua* linguistic objects—and not just used to refer to objects in the world; secondly, a mechanism of ‘unquoting’, represented in (7b) by the square brackets […], which suspends the quotation.

In other words, according to the analysis proposed by Maier, FID is a direct discourse where some elements of the quoted discourse are unquoted. Which elements in the sentence get unquoted is a matter of pragmatics. It is still poorly understood which pragmatic principles govern the availability of the unquotation mechanism. What needs be done—in Maier’s view—is a case-by-case analysis of the pragmatic factors that motivate the limited amount of unquotations in FID.

## Comparison between the two analyses

In 1.2 we have considered a number of arguments that have been raised against the analysis proposed by Schlenker (2004). In this section we look at some of these arguments in some more detail.

We begin with the argument regarding gender in FID. We have seen above that gender features as expressed on pronouns are actually interpreted relative to the context of thought, rather than to the context of utterance. In which sense is this an argument against Schlenker? We think it shows that the reason why pronouns are interpreted in the context of utterance cannot consist in the fact that pronouns (as well as tenses) are variables. In fact, once pronouns are decomposed into bundles of grammatical features (gender, number, person), it should rather be emphasized that some of these features (gender and, as we will see in a moment, number) are interpreted relatively to the context of thought (CT), whereas other features, namely person, are interpreted relatively to the context of utterance (CU). To see this in some detail, consider again the example discussed above, in a slightly different version. Suppose Mary was addressing Robin and, therefore, was using a second person pronoun in her original speech:

(7) [Mary was talking to Robin, who she believes to be a man, but who is actually a woman]
  Where had he been all morning, for instance? Mary asked her.
  (from Maier (2015))

Here, it is important to notice that the pronoun originally used in Mary’s speech is ‘you’ and not ‘he’. If we had reported Mary’s speech as an instance of direct discourse, we would have got: “Where have YOU been all morning, for instance?” This observation clearly shows that if the person presupposition encoded by the pronoun we have in FID (i.e. ‘HE’) had to be satisfied in CT, it would fail, since a third-person feature is not able to refer to the addressee in CT. It follows that the third-person feature in ‘he’ is satisfied in CU, where the intended referent (i.e. Robin) is neither the addressee nor the speaker.

From Maier’s perspective, this means that pronouns should be decomposed into feature bundles, where each feature, having an independent semantic value, can be quoted or unquoted independently [an analysis originally suggested by Doron (1990)]. However, there is an important sense according to which feature decomposition might work as well for Schlenker: gender features behave as demonstratives and referential indexicals (they are evaluated in CT), whereas person features behave as tenses (they are evaluated in CU). It is thus worth emphasizing that, once feature decomposition is assumed, the behavior of gender in FID is not, in itself, a decisive argument in favor of Maier’s ‘mixed quotation’ approach. Nor is it a decisive argument against Schlenker’s distinction between CU and CT. Indeed, what is lost of Schlenker’s original account is the idea that pronouns and tenses depend on the context of utterance because they denote variables. Once feature decomposition is assumed, what needs to be explained is what distinguishes *person* and *tense* from other morphological features.^4^

Before continuing, let us briefly see that number behaves as gender in FID: suppose Mary is actually married to two husbands without knowing it (one might think of one of the canonical ‘comedy of the errors’ scenario’s, where two male twins actually alternate in Mary’s life, while she believes it’s always the same person). Consider now the scenario and the instance of FID in (8):

(8) [Recently Mary observed that her husband Robin behaves rather inconsistently, without knowing that this inconsistency is due to the fact that two persons, Robin and Robert, play the role of her husband]
  a. Tomorrow she would ask HIM how HE could behave so strangely – Mary thought
  b. # Tomorrow she would ask THEM how THEY could behave so strangely – Mary thought

Notice that it is not the case that plural number, in this sort of settings, is generally excluded. Given the proper context, plural is readily permitted in indirect discourse. Suppose for instance that we had to rephrase the FID in (8a) into indirect discourse. The outcome in (9) would be a perfectly natural option, an option in which singular number, as it would occur in direct discourse, is turned into plural number:

(9) I am very concerned about what might happen to Robin and Robert. I understood Mary intends to ask THEM how THEY could behave so strangely

On these grounds, we conclude that when a second-person pronoun (‘you’) is used in direct discourse, the reason why it cannot be maintained in FID is that person features (contrary to gender and number features) amount to presuppositions that cannot be satisfied in CT. Crucially, the analysis of how pronouns behave in FID involves feature decomposition, but this is a conclusion to be drawn independently of the analysis that is ultimately adopted.

The second argument raised against Schlenker’s analysis concerns the use of proper names in FID. Maier provides evidence that not only pronouns and tenses are evaluated relatively to CU, but also proper names can be evaluated relatively to the CU. We believe that the facts concerning proper names in FID are significantly more complex. In particular, it is not the case that proper names can be used transparently in FID. Consider the following example as well as the two possible continuations in (10a) and (10b):

(10) Bill had become friend with Sam, who had recently moved in the neighborhood. What Bill didn’t know was that Sam’s real name was not Sam, but Moriarty, a dangerous criminal using that anonymous neighborhood as a safe place to hide from the police. Later that afternoon, Bill took the phone and dialed Sam’s number:
  a. Would Sam like to join him for dinner, he asked the moment Sam picked up the phone.
  b. #Would Moriarty like to join him for dinner, he asked the moment Sam picked up the phone.

Notice how, in the given scenario, (10a) is a sound continuation, whereas (10b) is not. The examples strongly suggest that there is a further condition to be fulfilled in order for proper names to be used correctly in FID: Informally, a proper name can be used in FID only if it is recognized as a valid name for its referent by the person whose words or thoughts are reported. In Schlenker’ terms, this can be accommodated by assuming that proper names can be used at the condition that their referential function is recognized in CT: The protagonist must be aware of the referential function of the name; that is, the epistemic state she is in must be one which allows her to use that name in order to refer to the purported referent. In example (10b), ‘Moriarty’ cannot be used felicitously because Sam does not recognize ‘Moriarty’ as a valid means to refer to the individual Sam; that is, the name ‘Moriarty’ is not recognized in CT as referring to the same individual as the name “Sam”.

On the basis of this and similar examples, we conclude that the use of proper names in FID is considerably less transparent than is claimed by Maier.^5^ In fact, not only proper names do not offer a decisive argument against Schlenker’s proposal, rather their behavior in FID can actually be successfully described by relying precisely on the distinction between CT and CU that Schlenker proposes.

To summarize, we believe that there are theoretically more or less plausible ways to defend Schlenker’s proposal against some of the arguments put forward by Maier and Reboul. In particular, we have seen that: (i) the behavior of gender features in FID can be accounted for by assuming that pronouns are decomposed into independent morphological features, as independently maintained by Maier; (ii) proper names (as well as definite descriptions) can replace a pronoun in FID, but only at the condition that their referential function applies in CT. At least as far as these aspects are concerned, we conclude that it is possible to modify Schlenker’s theory in ways that allow it to achieve the same empirical adequacy as Maier’s proposal.

More generally, however, we would like to conclude this section by pointing out a common weakness of Schlenker’s and Maier’s approaches. Schlenker’s original proposal tries to provide a principled explanation of the distinction between pronouns and tenses, on the one hand, and other indexicals, on the other hand, on the basis of the observation that pronouns and tenses are ‘variables’. As we saw, this explanation cannot be maintained once feature decomposition is assumed. What needs to be explained, then, is what principled reasons determine that only person and tense *features* are evaluated relatively to the context of utterance. Maier’s proposal, on the other hand, faces a similar problem. Maier claims that the mechanism of unquotation is governed by pragmatic principles: Which elements in the sentence get unquoted is a matter of pragmatics. However, Maier points out, it is still poorly understood which pragmatic principles govern the availability of the unquotation mechanism. What needs be done—in Maier’s view—is a case-by-case analysis of the pragmatic factors that motivate the limited amount of unquotations in FID, especially in order to understand why they are primarily restricted to tense and to the person features of pronouns. We conclude that, while both theories (with the provisions discussed above) provide an empirically adequate account of the semantics of FID, they both fail in providing a principled explanation of what can and what cannot be evaluated from the perspective of the actual speaker.

## Proposal: FID as phenomenal identification

The insight that we would like to put forward is that FID is an indirect discourse report in which the Higher Experiencer (the experiencer of the context of utterance, henceforth HE) reports the experience associated with the Lower Experiencer (the experiencer of the context of thought, LE) by ‘phenomenally identifying’ with this experience.

From this perspective, FID is different from both direct and indirect discourse. Let us adopt, for solely descriptive purposes, Schlenker’s distinction between CT—the original context of the reported thought or speech, including its time, its author, etc., and CU—the context at which the original thought or speech is reported.

Direct discourse is represented by a main clause that is intended to literally reproduce the context of thought, by reproducing what the protagonist said or thought. The context of utterance of a super-ordinate clause only serves to introduce the report as a ‘quote’, that is, to signal that the context of thought is not to be identified with the context of utterance (the time at which the speech or thought is interpreted is not the time at which the speech or thought is produced) and to signal that there is a second utterer besides the author in the context of thought, that is, a *different experiencer* that reproduces the original speech or thought at a *different time*.

The benchmark of direct discourse is that there is no interpretive interference between context of thought and context of utterance besides making clear that the context of thought is not intended as featuring a presently developing thought but as featuring a ‘quoted’ thought.

On the other hand, FID is also different from indirect discourse. Indirect discourse not only makes clear that there is a context of utterance distinct from the context of the original thought, it also crucially shifts the interpretive mood proper to the context of thought into the interpretive mood proper to the context of utterance. This means that though the reported propositional content is intended to reproduce the content of the utterance that took place at a given context of thought, all the linguistic expressions that are used to convey this content are interpreted with respect to the *actual* context of utterance. This entails that what a protagonist said at a given context of thought is now interpreted *from the perspective* of a different speaker.

In a nutshell, we can say that direct discourse maintains context of thought and context of utterance completely distinct, whereas indirect discourse conflates them into a single context. Maier’s insight is that FID is a form of mixed quotation: context of thought and context of utterance are kept separated (as in direct discourse), but with a limited amount of pragmatically motivated exceptions. From this perspective, FID would amount to an ‘imperfect’ form of direct discourse. This would explain why virtually all linguistic and metalinguistic indicators point towards direct discourse.

We contend that this is a serious mistake. Pointing to essential similarity with direct discourse does not capture the fact that FID expresses the *empathy* of the Higher Experiencer (the agent in CU) with the Lower Experiencer (the agent in CT). On the contrary, direct discourse is an emotionally neutral reproduction of the content produced in the context of thought. The insight that we wish to put forward is that FID is simply a way to change this condition, that is, FID is a way to express empathic identification, from the part of the utterer in CU, with the experience expressed in CT, by means of adopting the *phenomenal perspective* of the protagonist in CT. Before discussing the evidence that supports this interpretation of FID, it is important to notice that indirect discourse is not suited to achieve empathic identification. In fact, it is intuitively fair to claim that indirect discourse simply reinterprets what was originally expressed in the original context of thought from the new perspective of the context of utterance, without any inherent commitment to empathic identification.

Consider the following fragment, discussed by Reboul (2013), from Modiano’s novel *Accident nocturne*:

(13) I drew out of my pocket the “report” I had signed. So she was living in the square de l’Alboni. I knew that place because I had often got down at the nearest underground station. No problem that the number was missing. **With the name: Jacqueline Beausergent,*I*would manage**

In this novel, the protagonist (who is also the narrator, that is, Modiano) is looking for a young woman who has run him down in her car. The address on the “report” is incomplete, but the protagonist thinks he will “manage” to find her nevertheless. However, in the novel, we discover he does not find the young woman and the narrator (the present time Modiano who is writing the story) knows that he won’t. In other words, we have here a situation in which Modiano (the referent of the first-person pronoun “I” in the FID fragment) finds Jacqueline Beausergent only relatively to CT and, crucially, not relatively to CU.

The use of the first-person pronoun in (13) exactly parallels the use of the third-person pronoun in (11), since these are pronoun occurrences in which the pronoun is interpreted in CT and not in CU. However, (13) introduces a further complication. In (13), a first-person pronoun is used in FID to refer to the protagonist, that is, to LE. According to the original intuitions about FID expressed in Banfield (1982), this should never happen. Banfield claims that in contexts like (14) the first-person pronoun cannot be interpreted as referring to the HE (i.e. as referring to the narrator) but is only licensed under the condition that the narrator also is the addressee in CT.

(14) [situation, roughly: protagonist (she) thinks the narrator is a very nice guy]
  a. #Oh how extraordinarily nice I was! she thought
  b. Oh, how extraordinarily nice I was! she told me

As noticed by Schlenker, the licensing condition on first-person pronouns is in fact looser than suggested by Banfield. In (15), the referent of “I” in FID is not the addressee in the context of thought, but this occurrence of “I” is nevertheless felicitous (the question is of course why):

(15) [situation, roughly: protagonist (she) thinks the narrator is a very nice guy]
  Oh how extraordinarily nice I was, she told my father, without realizing that I was listening to their conversation.

Here, at least two questions arise: (i) Which licensing condition is operative in (15)? (ii) Why is the first-person pronoun in (13) allowed to refer to the narrator or, as argued by Reboul, to the thinker in CT (remember that “I” should be licensed, according to Banfield, only in contexts where it refers to the *addressee* in the context of thought)?

In what follows, we will see how these puzzles may be solved by means of the following ingredients: (i) FID involves *phenomenal identification*; (ii) Schlenker’s distinction between CT and CU plays a significant theoretical role (*contra* Maier); (iii) the interpretative behavior of pronominal features and tenses with respect to CT and CU depends on the properties of phenomenal identification; (iv) the eccentric behavior of first-person pronouns and proper names in FID depends on the conditions regulating the relationship between CT and CU, as imposed by the nature of FID as a process of phenomenal identification.

If FID expresses phenomenal identification with LE from the part of HE, it follows that the propositional content encoded by FID should be presented from the perspective of LE. Since HE intends to rebuild the reported experience in herself, adopting a perspective different from that of LE would not do. We propose that this property of FID as phenomenal identification derives the first and main property of FID, formulated in (16):

(16) All linguistic expressions in FID are compulsorily interpreted relatively to CT

What (16) is purported to capture is the fact that all linguistic and metalinguistic indicators in FID point to FID as a variant of direct discourse, in which interpretation takes place relative to the context in which LE is the speaker/thinker. In fact, as Maier demonstrates, the effects of (16) are extremely pervasive: Expressives, demonstratives, and referential indexicals are all interpreted in CT, as well as grammatical features of pronouns such as gender and number, as we have shown above. One of the consequences of (16) is thus that there is no real symmetry between pronouns and tenses, *pace* Schlenker (whose suggestion, remember, was that pronouns and tenses are interpreted in CU because they are variables). As we have seen, in fact, pronouns have to be treated as feature bundles.

Let us then reformulate the main question addressed in the present article:

- (i) Why should person and tense not be interpreted in CT, providing an exception to the general validity of (16)?

We contend that this follows from the ontological status of the process of phenomenal identification. In direct discourse, a speaker/narrator simply reports some propositional content from the perspective of a distinct experiencer (LE, according to the terminology adopted here). Conversely, in FID the ontological requirement that need to be satisfied in order for phenomenal identification to take place is that there be two distinct experiences and two distinct experiencers. HE is the experiencer_1_ of experience_1_ (taking place at t_1_) that is intended to be very similar (ideally, up to full identification) to experience_2_ as lived by experiencer_2_ (LE) at t_2_, with t_2_ distinct from t_1_. Suppose now that *all* linguistic expressions in FID are interpreted relative to CT. How could one mark a difference with respect to direct discourse? Or more exactly, how could one tell that there is an attempt by HE to rebuild in herself the experience lived by a distinct experiencer (LE) rather than to provide an emotionally neutral report of what LE said or thought? Suppose further that this entails that some linguistic expressions have to be interpreted from the perspective of HE (that is, relative to the context of utterance) in order to signal that the original thought or speech is not simply reported, but rather dynamically put in relation, in the cognitive and emotional terms associated with phenomenal identification, with the context of utterance. From this perspective, the real question boils down to the following:

- (i) Which linguistic items qualify as optimal candidates for interpretation in CU?

Remember that, ontologically, we have to grant the existence of two distinct experiencers and two distinct experiences. Delfitto and Fiorin (2014b) provide linguistic evidence that the *de se* reports of certain control verbs (such as “imagine” and “remember”) are also unambiguously immune to ‘error through misidentification’ on the part of the author of the reported attitude. As originally discussed by Higginbotham (2003), the statement “Jim remembers saying that John should finish his thesis by July” cannot be true in a scenario in which John fails to identify himself as the person having the experience of saying that John should finish his thesis by July. In fact, the sentence is judged as unsound in a context such as “Jim remembers saying that John should finish his thesis by July, although, now that he thinks about it, he is not sure it was him who he remembers saying it.” Notice, importantly, that the immunity to error is not conveyed by slightly different grammatical formats. The sentence “Jim remembers himself saying that John should finish his thesis by July” does not contradict the following continuation: “although, now that he thinks about it, he is not sure it was him who he remembers saying it.” Delfitto and Fiorin (2014b) contend that a proper modeling of the semantics of these peculiar statements requires that experiencers and experiences be regarded as belonging to ontological classes distinct from individuals and events. In particular, two properties are of importance in the context of the present discussion: (i) for each experience there is one and only one experiencer (hence, any two experiences with two different experiencers are necessarily different experiences); (ii) for each experience there is one and only one point in time at which the experience occurs (hence, any two experiences occurring at two different points in time are necessarily numerically distinct experiences). While we refer the reader to Delfitto and Fiorin (2014b) for a throughout discussion of the precise metaphysical status of these ontological classes and their consequences for a theory of meaning, in the reminder of this article we would like to demonstrate how the two properties mentioned above provide a principled answer to the puzzle of FID.

Let us return to our main question (i): *Which linguistic items qualify as optimal candidates for interpretation in the context of utterance?* Our answer is that it suffices to link each of the two experiences to two distinct times and two distinct experiencers: this is achieved by interpreting tense and person in the context of utterance. After all, this is precisely what a process of Phenomenal Identification entails: An agent attempting to reproduce the subjective phenomenal experience of another agent. Ideally, full identification produces two experiences that are equal in all regards except in that they are experienced by two distinct experiencers and occur at two distinct points in time (i.e. they are *qualitatively* identical though *numerically* distinguished).

As for the experiencers, remember that the semantics of first-person is strictly associated to self-ascription (see Wechsler 2010). A sentence in which one of the arguments is a first-person pronoun encodes the interpretation according to which this argument not only corresponds to one of the participants of the state or event portrayed, but also corresponds to the bearer of the relevant propositional attitude in CT. In other words, a first-personal sentence is a sentence in which one of the participants in the state/event portrayed is also the experiencer of the mental event (speech or thought) whose content is the portrayed state/event. From the present viewpoint, we might say that one of the participants is the agent from whose perspective the relevant ‘experience’ is reported. This becomes particularly clear if we adopt the view according to which a first-personal sentence expresses a property that need be self-ascribed by the speaker, and a second-personal sentence expresses a property that need be self-ascribed by the addressee (cf. Wechsler 2010). This view entails that when one listens to a first-personal sentence uttered by someone else, the only way to assess the truth-value of this sentence consists in identifying with the mental state of the speaker. Similarly, the only way to assess a second-personal sentence when one produces it (instead of listening to it) is for the speaker to identify with the mental state of the addressee.

Let us see what the consequences are for the use for the person-features in FID. If we use a first-person pronoun in a FID-context, and we interpret the person feature in CT, there is no way to avoid that the property expressed in FID be interpreted as self-ascribed by the protagonist. Consider an example, as in (17):

(17) #How intelligent I was! – Elena thought

Interpreting “I” in (17) in CT entails that “I” necessarily refers to the experiencer in CT (i.e. Elena). One sees thus that this would make (17) completely equivalent to an instance of direct discourse of the kind: “Elena thought: ‘How intelligent I am!’” On the other hand, if we interpret the first-person feature relative to CU, “I” would refer to the utterer of (17), that is, to HE. Why is this infelicitous? From the present perspective, the answer is straightforward: if “I” is interpreted in CU, (16) is violated, since “I” would be a linguistic expression that is not interpreted in CT. This is incompatible with FID and this is the reason why (17) is infelicitous. On these grounds, we conclude that we have a principled reason why first- and second-person pronouns must be shifted to third-person (or to names; see the discussion above) in FID: The semantics of first/second person is simply *incompatible* with the nature of FID as encoding phenomenal identification. Of course, we have also seen in the preceding section that there are exceptions to the ban on first-person in FID. It is to these exceptions that we have to turn now.

Before doing that, however, there is a potential objection to the proposed account of Person in FID that we should consider at this point. One might say that if “I” cannot be interpreted in CU because of (16), the same reasoning should be applied to tense, that is, interpreting tense relative to CU should be equally infelicitous. However, there is a straightforward answer. Interpreting “I” in CU and in CT delivers two referentially distinct individuals (since, normally, HE is distinct from LE, that is, the protagonist is different from the narrator; exceptions will be considered below). Conversely, interpreting tense at CU and in CT does not deliver two referentially distinct times. To see this, just consider the minimal pair in (18):

(18) a. Mary thought: “I will tell him to leave” (direct discourse)
  b. Mary thought that she would tell him to leave (indirect discourse)

Based on a Reichenbachian semantics for tense, (18b) is usually referred to as ‘future in the past’: Mary’s saying takes place after Mary’s thought, but not necessarily after the time at which (18b) is uttered. The difference between the two tenses in (18) reduces to differences in the relational values of the relevant utterance times, event times and reference times. In absolute terms, however, interpreting the tense in the context of thought [as is the case in (18a)] or in the context of utterance [as is the case in (18b)] does not deliver two distinct times, rather it delivers, in absolute terms, the very same time along the time axis. It is worth noticing that these considerations not only provide an answer to the potential objection formulated above. They also make clear that there is a sort of *uniformity condition* operating on FID: The interpretive result that we get when we shift the interpretation of a linguistic item or grammatical feature in FID from CT to CU must preserve, in some sense to be defined, some essential aspects of the interpretation that we would get by interpreting that very same item or feature in CT. In the case of tense, as we have seen, uniformity can be understood as *numerical identity in extensional terms*. The natural question is what this means in the case of Person. Significantly, answering this question is tantamount to deriving the eccentric features of first-person and names in FID that we have reviewed above.

Let us start with Reboul’s example from Modiano, reproduced below as (19). One of the problems that this example raises for the traditional analysis of FID (as Banfield’s) is that the first-person pronoun appears to be perfectly legitimate. Why?

(19) I drew out of my pocket the “report” I had signed. So she was living in the square de l’Alboni. I knew that place because I had often got down at the nearest underground station. No problem that the number was missing. **With the name: Jacqueline Beausergent,*I*would manage**

What distinguishes (19) from the cases in which a first-person pronoun is excluded is the fact that the situation described is one in which the narrator phenomenally identifies with an experience that he himself lived, at a preceding time. So, though there are two distinct experiences (the one Modiano is in at the moment he writes and the one he was in at the moment he was looking for the woman who had run him down) and two different experiencers (Modiano as the narrator at t_1_ and Modiano as the protagonist at t_2_), the narrator and the protagonist are *extensionally identified*. Since the individual referred to by “I” is—extensionally—the same individual in CT and in CU, it is fair to claim that shifting the interpretation of “I” from CT to CU does not violate the *uniformity condition* that we have argued is operative in the case of tense. Conversely, this is clearly not so in cases such as (17): here, shifting “I” from CT to CU involves a radical referent change [since Elena and the narrator are clearly referentially distinct in (17)].

However, there are reasons to think that in the case of Person, the application of the uniformity condition results in an interpretive requirement that is weaker than extensional identity between the referent of “I” in CT and the referent of “I” in CU. Consider in this respect (14b), reproduced here as (20):

(20) **Oh, how extraordinarily nice*I*was!** She told me

Under the working hypothesis that we are defending here, according to which FID expresses phenomenal identification between narrator and protagonist, the narrator in (20) is trying to identify with the experience lived by the female protagonist. Clearly, the referent of “I” in CT is the female protagonist, whereas the referent of “me” in the context of utterance is the narrator, a distinct character. There is thus no extensional identity between the referent of the first-person pronoun in the context of thought and in the context of utterance. Strikingly enough, however, the first-person pronoun is perfectly legitimate in (20) as referring to the narrator, in spite of the fact that uniformity, as defined above, is violated. Again, the question is why this is so.

We think that this question also admits a principled answer, which eventually elucidates the actual conditions of use of first-person in FID. Here is the basic insight. There is a sense according to which “I” in (20), interpreted in CU as referring to the narrator, retains a first-personal interpretation in CT. It is in this sense—we claim—that *uniformity* is satisfied in the shift from CT to CU. To see why this is the case, consider the fact that, as observed by Banfield, the role of the narrator in the context of thought is that of addressee. In other words, mapping FID into direct discourse would deliver a sentence like “How extraordinarily nice you are!”, where ‘you’ refers to the narrator. This means that in CT the referent of “I”—the narrator—is the addressee of the sentence “How extraordinarily nice you are!”. In terms of self-ascription, this entails that the narrator, as the addressee in context of thought, self-ascribes, in the context of thought, the property ‘λx. x is extraordinarily nice’. This amounts to a sort of *de se* effect in CT, relative to the referent of “I” in the context of utterance, i.e. the narrator. In CT, the narrator undergoes a first-personal interpretation: he is not simply the individual of whom the property ‘λx. x is extraordinarily nice’ holds, he is also the individual who is aware of the fact that this property holds of himself. This is the reason why we speak of a ‘*de se* effect’. And this is the reason—we submit—why *uniformity* is satisfied in (20): the narrator operates as a center of consciousness (that is, in a first-personal way) both in CT and in CU.

This interpretation is immediately confirmed by Schlenker’s observation that a first-person pronoun is licensed in FID not only in cases such as (20), where the referent of “I” is the addressee in context of thought, but also in cases such as (21), in which the referent of “I” is not the addressee (the addressee is the narrator’s father):

(21) [situation, roughly: protagonist (she) thinks the narrator is a very nice guy]
  **Oh how extraordinarily nice*I*was**, she told my father, without realizing that I was listening to their conversation

From the present perspective, this kind of extensions in the legitimate usage of the first-person pronouns in FID loses all its puzzling aspect. In fact, notice that though the narrator is not the addressee in (21), it is the narrator who self-ascribes the property ‘λx. x is extraordinarily nice’. Mapping FID in (21) into direct discourse would deliver a sentence like “How extraordinarily nice [narrator’s name] is!” In CT, the narrator listens to this sentence, and is aware of the fact that the referent of the narrator’s name is he himself. For the narrator, the sentence is thus equivalent, in CT, to the sentence “How extraordinarily nice I am!”, whereby the narrator self-ascribes the property ‘λx. x is extraordinarily nice’. Again, the narrator operates as the center of consciousness both in CT and in CU, the uniformity condition is satisfied and the first-person pronoun is perfectly felicitous as it occurs in FID.

These ideas lend themselves in a natural way to a solution of the puzzles that we have discussed in the preceding section with regard to the use of proper names in FID. The two main issues revolve around the reasons why (i) sometimes a name is chosen instead of a third-person pronoun when mapping from CT to CU takes place in FID; (ii) the chosen name must be recognized by the person whose speech or thought is reported (LE) as a valid name for the intended referent. Let us start with (ii), i.e. the ‘transparency condition’ on proper names. A case in point is the contrast, reproduced below, between (22a) and (22b).

(22) [Mrs Cabot—the person whose words are reported—recognizes Arnie’s voice. However, Arnie is not known to her with the name “Arnie”, but with the name “Ortcutt”]
  a. #**Arnie** had had his last chance with her, that voice said, and he had blown it
  b. **Ortcutt** had had his last chance with her, that voice said, and he had blown it

The difference between (22a) and (22b) can be elucidated in terms of a *de se* effect, present in (22b) and absent in (22a). In (22) Mrs Cabot is aware in CT of the fact that the person she is talking to is named Ortcutt, whereas she is not aware that this person is (also) named Arnie. It follows that the semantics of the second-person pronoun that would occur in direct discourse (“You’ve had your last chance with me, and you’ve blown it”) can be reconstructed only at the level of CU in (22a), where the narrator is aware of the extensional identity between Arnie and Mrs Cabot’s interlocutor, whereas it can already be reconstructed at the level of CT in (22b), where Mrs Cabot herself, as the protagonist, is aware of the identity between Ortcutt and her interlocutor. We conclude that (22b) provides a context in which mapping a second-person pronoun into a name (activating the context of utterance as a further interpretive layer) does not destroy the original second-person semantics in the context of thought: Under the detected *de se* effect, not only the narrator but also the protagonist would be in condition to recover the original content of the protagonist’s speech. This condition is not satisfied in (22a), where only the narrator can rebuild Mrs Cabot’s original speech. We propose this is a *uniformity violation* (in the sense defined above) and that this is thus the reason why FID is infelicitous in (22a).

Consider next the issue concerning the choice of a proper name (instead of a third-person pronoun) in FID. A case in point is reproduced below as (23):

(23) Bill and Eric were fighting, when Sookie stepped between them. **Did*Bill*really think*he*could challenge*his*boss like that?** she demanded, before turning to Eric. **And what the hell was HE thinking?**

From the narrator perspective (i.e. from a context of utterance perspective) the use of a third-person pronoun in place of the underlined occurrence of “Bill” in (23) would be referentially opaque: Is Sookie addressing Bill or Eric in the given context? On the other hand, (23) also shows that once a name has been used to circumvent referential opacity, FID can be continued by using a third-person pronoun. A similar case, where the proper name stands for a first-personal original thought, is shown in (24) below [drawn from Reboul et al. (2015)]. Here, given previous mention of Mrs. Wix, the use of a third-person pronoun (‘she’) would turn out ambiguous on the side of the narrator:

(24) The only mystification in this was the imposing time of life that her [Maisie’s] elders spoke of as youth. For Sir Claude then Mrs. Beale was “young”, just as for Mrs. Wix Sir Claude was (…). *What therefore wasMaisie herself, and, in another relation to the matter, what therefore was mamma?* (James 2011 (1897), loc. 51112).

## Conclusions

In this contribution, we have proposed that the need for additional semantic parameters in the interpretation of FID (mainly the split between a Context of Thought and a Context of Utterance) stems from the nature of FID as encoding a linguistic process of phenomenal identification. We have shown how this analysis provides evidence for the sensitivity of higher-order cognitive systems such as language to an ontology of experiences, whereby two distinct experiencers end up sharing the same qualitative experience, based on two numerically distinct experiences. In this way, the analysis of FID sheds new light on the rich cognitive and linguistic interplay that characterizes first-personal interpretations in natural language. Quite interestingly, and decisively for the particular purposes that we intended to pursue here, we have argued that not only the nature of FID as intermediate between Direct and Indirect Discourse, but also many of the most puzzling properties of FID (including the elusive similar behavior of Person and Tense) can be derived as a linguistic reflex of some basic cognitive requirement associated with the process of phenomenal identification. Last but not least, we have tried to offer, on many points, a fine-grained analysis of some of the most debated linguistic properties of FID.

## Footnotes

1 For a recent overview, see Reboul et al. (2015).

2 However, it should also be noticed that Banfield’s analysis reduces FID to a form of direct discourse.

3 For a recent analysis of indirect reports, effectively combining semantic and pragmatic considerations, see Capone (2010, 2016).

4 It is interesting to observe that independent arguments can be presented in favor of the idea that person features are evaluated relative to context of thought whereas gender features are evaluated relative to the context of utterance. Basing himself on a sample of more than one hundred languages, Baker (2008) observes that subject-verb agreement is the only configuration that licenses person-agreement. Baker makes sense of this data by proposing the Structural Condition on Person Agreement (SCOPA), which basically restricts person-agreement to strictly local configurations. In order to account for SCOPA, Baker proposes that Person inherently triggers an A-bar dependency. The reason is that person features are not inherently generated on the agreeing noun but are basically induced from outside by means of a sort of operator-variable agreement (see also Kratzer 2009; Delfitto and Fiorin 2011, 2014a: that is, person features are base-generated at the clause level and copied by Agree on the agreeing noun phrase by means of an operator-variable dependency (that is, an A-bar-dependency). The picture that emerges is one whereby gender features are base-generated on NPs whereas person features are base-generated in a dedicated position in the left periphery of the clause; they are transmitted to NPs by means of Agreement. Person features, syntactically, are more similar to Tense than to gender features. There is a semantic counterpart to these syntactic considerations. In some languages, first and second person features can be interpreted relatively to a reported context of utterance (Anand 2006; Anand and Nevins 2004; Delfitto and Fiorin 2011, 2014a; Schlenker 1999, 2003). In such languages, the counterpart of the English sentence *John said that I am a hero* is ambiguous between two interpretations: (1) John said that the speaker is a hero; (2) John said that John is a hero. In the second reading, *I* is interpreted as the speaker in the ‘reported’ speech act. These languages show that the interpretation of person features can be manipulated by scope taking operators. The same does not hold for gender features. Cooper (1983) analyzes gender features as introducing presuppositions that he calls “indexical”, in the sense that they must always be resolved globally (cf. Yanovich (2010) for some relevant qualifications). To see how this is intended to work, compare the sentence *Bill said that the Queen of France talked for two hours*, where the presupposition of the complement of ‘said’ need not project to the actual world, with *Bill said that she talked for two hours*, where the gender presupposition of ‘she’ must project to the actual world.

5 Similar considerations can be made for referential uses of definite descriptions. Let us imagine that Bill became acquainted with the man living next door, unware of his identity with a very suspicious man Bill saw late at night wearing a brown hat. In this scenario (1) seems an appropriate way to report in FID Bill’s request to invite his neighbor for dinner. On the other hand, (2) is not sound, in spite of the fact that the reader is aware of the identity between the referents of the two descriptions.

1. Would his neighbor like to join him for dinner
2. Would the man in the brown hat like to join him for dinner.
